# Cross-education and detraining effects of eccentric vs. concentric resistance training of the elbow flexors

**DOI:** 10.1186/s13102-021-00298-w

**Published:** 2021-09-06

**Authors:** Shigeru Sato, Riku Yoshida, Ryosuke Kiyono, Kaoru Yahata, Koki Yasaka, Kazunori Nosaka, Masatoshi Nakamura

**Affiliations:** 1 Institute for Human Movement and Medical Sciences, Niigata University of Health and Welfare, 1398 Shimami-cho, Kita-ku, Niigata 950-3198 Niigata, Japan; 2 Department of Physical Therapy, Niigata University of Health and Welfare, Niigata, Japan; 3 School of Medical and Health Sciences, Edith Cowan University, Joondalup, WA Australia

**Keywords:** Cross-transfer effect, Elbow flexors, Muscle strength, One-repetition maximum, Muscle thickness, Maximum voluntary isometric contraction

## Abstract

**Background:**

Unilateral resistance training increases the strength of the contralateral non-trained homologous muscles known as the cross-education effect. We tested the hypothesis that unilateral eccentric resistance training (ET) would induce greater and longer-lasting cross-education effect when compared with concentric resistance training (CT).

**Methods:**

Young (20–23 y) participants were allocated to ET (5 males, 4 females) or CT (5 males, 4 females) group that performed unilateral progressive ET or CT of the elbow flexors, twice a week for 5 weeks (10 sessions) followed by a 5-week detraining, and control group (7 males, 6 females) that did not perform any training. Maximum voluntary isometric contraction torque of the elbow flexors (MVIC), one-repetition maximum of concentric dumbbell curl (1-RM), and biceps brachii and brachialis muscle thickness (MT) were measured from the trained and non-trained arms before, several days after the last training session, and 5 weeks later. A ratio between the trained and non-trained arms for the change in MVIC or 1-RM from pre- to post-training (cross-body transfer ratio) was compared between ET and CT groups.

**Results:**

The control group did not show significant changes in any variables. Both ET and CT increased (*P* < 0.05) MVIC (22.5 ± 12.3 % vs. 26.0 ± 11.9 %) and 1-RM (28.8 ± 6.6 % vs. 35.4 ± 12.9 %) of the trained arm without a significant difference between groups. MVIC was maintained after detraining for ET but returned to the baseline for CT, and 1-RM was maintained after detraining for both ET and CT. For the non-trained arm, MVIC (22.7 ± 17.9 % vs. 12.2 ± 10.2 %) and 1-RM (19.9 ± 14.6 % vs. 24.0 ± 10.6 %) increased similarly (*P* > 0.05) after ET and CT, and MVIC returned to the baseline after detraining, but 1-RM was maintained for both groups. An increase (*P* < 0.05) in MT was found only after ET for the trained arm (7.1 ± 6.1 %). The cross-body transfer ratio for MVIC was greater (*P* < 0.05) for ET (90.9 ± 46.7 %) than CT (49.0 ± 30.0 %).

**Conclusions:**

These results did not support the hypothesis and showed similar changes in the most of the variables between ET and CT for the trained and non-trained arms, and strong cross-education effects on MVIC and 1-RM, but less detraining effect after ET than CT on MVIC of the trained arm.

**Trial registration:**

University Hospital Medical Information Network Clinical Trials Registry (UMIN000044477; Jun 09, 2021).

## Background

Unilateral resistance exercise training increases muscle strength of not only trained muscles but also non-trained homologous muscles of the contralateral limb, which is known as the cross-education effect [15, 24, 26, 28]. A meta-analysis study concluded that the cross-education effect was observed irrespective of age, gender, and disease status, and the magnitude of increase in muscle strength of the non-trained muscle is 70–77 % of that of the trained muscle on average [15]. The cross-education effect may be useful for dealing with unilateral disorders due to fracture [23], ligament injury [17], and hemiplegia [11]. However, this may not be always the case, since Zult et al. [33, 34] reported that quadriceps resistance training of the non-operated leg of patients with anterior cruciate ligament (ACL) reconstruction did not accelerate recovery. The clinical utility of the cross-education effect has yet to be established, therefore it is essential to find an effective protocol of maximizing the cross-education effect in resistance training for rehabilitation in patients with unilateral disorders [14, 25].

One of the critical training variables that could affect the magnitude of the cross-education effect is muscle contraction types such as isometric, concentric, and eccentric contractions [5, 24]. It has been reported that eccentric resistance exercise training (ET) confers a greater cross-education effect than other contraction types [20, 22]. For example, Kidgell et al. [22] investigated the effect of unilateral ET in comparison to concentric resistance exercise training (CT) on the wrist flexor strength in young males and females. After 12 training sessions over 4 weeks, both groups exhibited a similar significant strength gain in the trained arm (ET: 62 %, CT: 64 %), but the extent of the cross-education effect was significantly greater for ET (47 %) than CT (28 %). The authors suggested that the greater cross-education effect of ET than CT was related to increased corticospinal excitability and reduced intracortical inhibition after ET. On the other hand, Tseng et al. [31] showed that the magnitude of cross-education effect on elbow flexor strength was similar (50 %) between ET and CT; however, the trained arm showed 50 % greater increase in maximal isometric contraction strength after ET than CT, inducing 50 % greater increase in elbow flexor strength of the non-trained arm after ET than CT. It has been reported that an intervention of 4–6 weeks is necessary to induce a cross-education effect [26]. Thus, the duration of the training period in the study by Tseng et al. [31] might have been sufficient, but the training frequency (once a week) might not have been enough to maximize the cross-education effect. Thus, it is interesting to increase the training frequency to twice a week to examine muscle strength changes of both trained and untrained arms after ET versus CT.

It is also important to know how long the adaptations produced by resistance exercise training could be maintained after detraining [27]. However, limited information is available for the effect of detraining after ET versus CT training on the trained and contralateral non-trained muscles. Coratella and Schena [8] compared the detraining effect of bench press exercise on pectoral muscles after ET (5 sets × 6 repetitions at 120 % of one repetition maximum: 1-RM), CT (6 sets × 7 repetitions at 85 % 1-RM), and traditional (concentric and eccentric) resistance training (4 sets × 5 repetitions at 90 % 1-RM) that were performed twice a week for 6 weeks. They showed that muscle strength and hypertrophy were maintained during the 6 weeks of detraining after ET, but not after CT and traditional resistance training. Housh et al. (1996a, b) investigated the detraining effect after ET or CT of the knee extensors in a separate study and reported that the muscle strength gained after 8 weeks of ET or CT was maintained similarly for the trained (ET: 100 %, CT: 93 %) and non-trained limb (ET: 81 %, CT: 87 %) after 8 weeks of detraining. It may be that the detraining effects are less prominent for lower limb than upper limb muscles, since the lower limb muscles are more actively involved in daily activities with relatively higher load [9]. Therefore, it is interesting to use upper limb muscles to examine the detraining effect after ET versus CT training not only for the trained but also for non-trained contralateral arm.

Therefore, the aims of the present study were to investigate the effects of unilateral elbow flexors ET and CT on muscle strength and muscle thickness of the trained and non-trained arms (cross-education effect) after training performed twice a week for 5 weeks with progressively increasing intensity, and detraining for 5 weeks. This study tested the following hypotheses: (1) ET would increase muscle strength in both trained and non-trained arms and muscle thickness in the trained arm greater than CT; and (2) ET would maintain muscle strength in both trained and non-trained arms, and muscle thickness in the trained arm greater than CT.

## Methods

### Participants and study design

A total of 31 (17 males and 14 females) healthy university students who were free from any orthopedic disorders of the upper extremity, had no history of previous neuromuscular and chronic diseases, and had not performed resistance exercise training or competitive sports in the past 6 months, participated in the present study. All participants were informed about the study purpose and procedures, and a written consent was obtained from each participant. This study was approved by the Ethics Committee of Niigata University of Health and Welfare (#18,305). The study was conducted in conformity with the policy statement regarding the use of human subjects by the Declaration of Helsinki.

The participants were randomly allocated to one of the three groups with considering the gender balance as follows; ET group (5 males, 4 females, age: 21.1 ± 0.9 y, height: 165.9 ± 7.7 cm, body mass: 58.4 ± 8.2 kg), CT group (5 males, 4 females, 20.9 ± 0.6 y, 167.2 ± 7.7 cm, 63.3 ± 10.8 kg) and control group (7 males, 6 females, 20.9 ± 1.9 y, 166.4 ± 8.9 cm, 57.8 ± 7.9 kg). No significant differences in the physical characteristics were evident among the groups. The effect size for changes in contralateral muscle strength after resistance training was reported to be 0.6 in a previous study [24]. Using this effect size, the sample size was estimated with an alpha value of 0.05 and a statistical power of 0.8 for the model of F-test, and the number of measurements was 3 (G*Power 3.1, Germany). This estimation revealed that 8 participants per group were sufficient.

For both groups, one arm was randomly assigned to training, and the other arm was served to examine the cross-education effect. Among the nine participants in each group, four participants used their dominant arm for the training, and the arm throwing a ball was defined as the dominant arm. All participants performed unilateral progressive resistance training for the trained arm twice a week for 5 weeks (10 training sessions in total) followed by 5 weeks of detraining. During the whole experimental period, participants were asked to refrain from any other form of strenuous physical activity than the training performed in the study. During the detraining period, participants were instructed not to carry a heavy object using arms. The dependent variables included maximal voluntary isometric contraction (MVIC) torque of the elbow flexors, concentric one-repetition maximum (1-RM) strength of the dumbbell curl, and muscle thickness of biceps brachii plus brachialis (MT). These variables were measured from both arms before (PRE), 3–9 days after the 10th training session (POST), and after the 5-week detraining period (De-Tr), and the changes over time were compared between the ET and CT groups. For the control group, each variable was measured in both dominant and non-dominant arms before and after 5 weeks without training. One of the arms was randomly considered as the trained arm, and the other was non-trained arm, when the control group data were compared with those of ET and CT groups in statistical analyses.

### Training protocol

Based on the training protocol used in the study by Tseng et al. [31], the training load was increased each week from 10 % (week 1), 30 % (week 2), 50 % (week 3), 80 % (week 4), and 100 % (week 5) of MVIC torque for the trained arm. In each session, six sets of five eccentric or concentric contractions (30 repetitions in total) were performed from 90° elbow flexion to 0° in the ET, or a slightly flexed (about 5°) to 90° elbow flexion in the CT, and the arm was returned to the starting position without the dumbbell for both training. The participants were seated on a preacher curl bench during resistance training, and the shoulder joint was 45° flexion, 0° abduction. If a participant had difficulty in controlling the dumbbell movement at a high intensity such as 80 and 100 % MVIC torque, the investigator assisted the participants for weaker elbow joint angles. The interval was 15 s between contractions and 2 min between sets. The maximum value of the three measurements was used to determine the dumbbell weight of each participant using the actual value (kg) obtained by a dynamometer (Mobie, Sakai Medical Co., Ltd., Tokyo, Japan). The dumbbell weight was readjusted by measuring the elbow flexion strength of the trained arm before the third, fifth, seventh, and ninth training sessions. Therefore, the number of the MVIC torque measures during the training period was 12 (3 attempts x 4 sessions).

Elbow flexors muscle soreness of the trained arm was quantified using a visual analog scale (VAS) that had a 100-mm continuous line with “not sore at all” on the left side (0 mm) and “very, very sore” on the right side (100 mm) when the elbow joint was actively extended maximally [4]. Muscle soreness was assessed before, immediately after, and 1 and 2 days after the 1st, 3rd, 5th, 7th, and 9th training sessions.

### Maximum voluntary isometric contraction (MVIC) torque

**Fig. 1:**
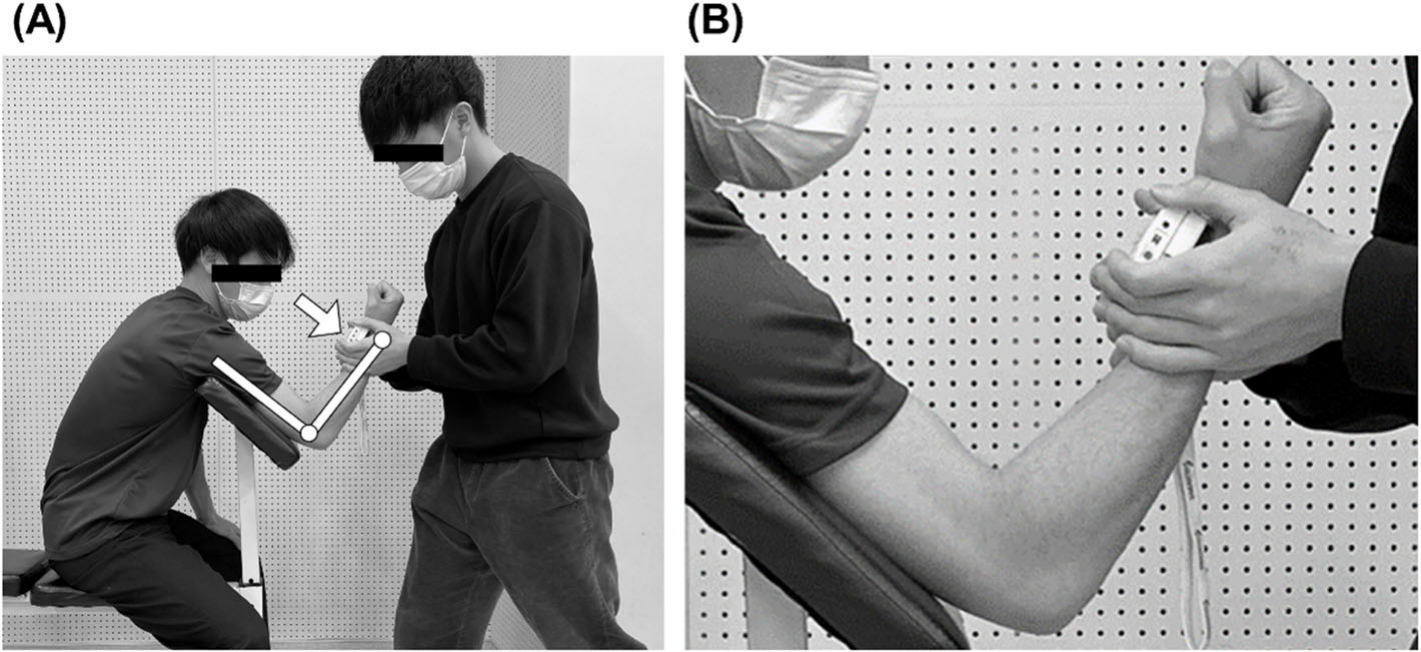
Measurement of maximum voluntary isometric contraction torque with a hand-held dynamometer (**A**) and its close look at the wrist where the hand-held dynamometer was placed (**B**)

The MVIC torque was measured by the hand-held dynamometer when each participant was seated on a preacher curl bench with the elbow joint at 90° flexion and the shoulder joint 45° flexion and 0° abduction (Fig. 1). The hand-held dynamometer was placed at the wrist, and each participant was instructed to flex the elbow joint maximally against the dynamometer for 3 s, and this was repeated three times with more than 30-s rest between attempts. The same investigator took all measures throughout the study. During the MVIC measurements, the investigator verbally encouraged the maximum effort of the participants. For each occasion, three measurements were taken with a 45-s rest between attempts, and the average value of the three measures was used for further analysis. The force (kg) measured by the dynamometer was converted to torque (Nm) by multiplying the gravitational acceleration (9.8 m/s^2^) and the forearm length.

### One-repetition maximum (1-RM)

1-RM of unilateral elbow flexion was measured using a dumbbell. Similar to MVIC torque measurements, each participant sat on a preacher curl bench at slight flexion of the elbow joint, and shoulder joint 45° flexion and 0° abduction. As a warm-up, participants performed 8, 5, and 2 repetitions with a dumbbell corresponding to approximately 40 %, 60 %, and 80 % of perceived 1-RM, respectively [29]. After the warm-up, 1-RM measurement was performed, and the initial weight was selected by each participant’s perceived 1-RM. The weight was increased by 0.5-1.0 kg until the participant could not lift the weight anymore through a full range of motion (elbow slight flexion position to the maximum flexion position) with the proper form, and 1-RM was identified within five trials. The interval between trials was 2 min, and the investigator verbally encouraged the maximum effort of the participants. The increment of the weight was 0.5 kg; thus its sensitivity was 0.5 kg.

### Muscle thickness (MT)

Muscle thickness (MT) of biceps brachii plus brachialis of the trained and non-trained arms was measured using B-mode ultrasonography with an 8-MHz linear probe (LOGIQ e V2; GE Healthcare Japan, Tokyo, Japan). The investigator minimized the pressure of the probe against the skin as much as possible. The measured site was 60 % of the distal line between the acromion and the lateral epicondyle of the humerus [2]. Each participant lay in the supine position on a bed with the arms placed at each side and forearm supinated while relaxing the arms. Muscle thickness was measured by the distance measurement function of the ultrasonography system. Ultrasound measurements of transverse-axis were repeated twice by the same investigator, and MT of EF was defined from the inner edge of the fascia to the humerus; the average value of the two measures was used for further analysis.

### Test-retest reliability of the measurements

The test-retest reliability of the MVIC torque, 1-RM, and MT measures was determined using 7 healthy students, using the measures taken one week apart without any training by coefficient variation (CV) and two-way mixed effect intraclass correlation coefficient (ICC_3, 1_). Their physical characteristics (5 males, 2 females, 21.3 ± 2.1 y, 173.7 ± 10.1 cm, 61.9 ± 9.1 kg) were similar to those in the ET and CT groups.

### Cross-body transfer ratio

A ratio between the trained and non-trained arms for the change in MVIC or 1-RM from pre- to post-training was calculated for each participant in the ET and CT groups by the following formula based on the previous study [15, 31].

$$Cross-body\;transfer\;ratio\;(\%)\;=\;(change\;in\;the\;non-trained\;arm\;from\;pre-\;to\;post-training\;/\;change\;in\;the\;trained\;arm\;from\;pre-\;to\;post-training)\;\times\;100$$

### Statistical analyses

SPSS (version 24.0; IBM Corp., Armonk, NY, USA) was used for the statistical analyses. The normality of the data was assessed by a Shapiro–Wilk test, which indicated that all variables were not normally distributed. Thus, non-parametric tests were applied to all variables. All dependent variables at baseline for the trained and non-trained arms were compared between the ET and CT groups by a Mann-Whitney U test. Changes in the variables from pre- to post-training and pre- to de-training in the trained and non-trained arm, respectively were compared for ET and CT groups separately by a Wilcoxon signed-rank test with Bonferroni correction. Also, changes in the variables before and after five weeks for the control group were compared by the Wilcoxon signed-rank test. The relative changes (%) in the variables from pre- to post-training were also calculated for the trained and non-trained arms, and comparisons among the groups were performed by a Mann-Whitney U test with Bonferroni correction. Effect size (ES) was calculated as a difference in the mean values between pre- and post-training or pre- and de-training divided by the pooled SD [6]. ES of 0.00–0.19 was considered trivial, 0.20–0.49 was small, 0.50–0.79 was moderate, and ≥ 0.80 was large. Mann-Whitney U test was used to compare the cross-body transfer ratio of MVIC torque and 1-RM between ET and CT groups from pre- to post-intervention. Spearman’s rank correlation coefficients (r_s_) were computed to examine the relationship between the trained non-trained arms for the changes in MVIC torque and 1-RM from pre- to post-training for each group separately and combining the data of ET and CT groups. The differences were considered significant at an α level of 0.05, and when the Bonferroni correction was applied, the differences were considered significant at an α level of 0.025 or 0.0167. Descriptive data are shown as mean ± SD.

## Results

### Baseline values and training

There were no significant differences between the ET, CT, and control groups for all variables at baseline (Table 1). All participants completed the training as planned and were able to perform six sets of five repetitions even for the higher intensity sessions. Mild muscle soreness developed after the 3rd (21.1 ± 15.2 mm) to 9th training sessions (11.1 ± 7.4 mm), and the highest VAS was observed after the 5th training session (33.6 ± 17.4 mm) for the ET group. On the other hand, muscle soreness after CT was minor, and the highest VAS was observed after the 5th training session (10.9 ± 13.9 mm), which was smaller (p < 0.01) than that of ET.

**Table 1:** Baseline values (mean ± SD, range) in maximal voluntary isometric contraction torque of the elbow flexors (MVIC), one repetition maximum of concentric dumbbell curl (1-RM), and muscle thickness of biceps brachii plus brachialis (MT) for trained and non-trained arms in the eccentric (ET) and concentric resistance training (CT) and control groups

		ET Group	CT Group	Control Group
MVIC (Nm)	Trained Arm	46.8 ± 20.2 24.0-80.1	43.9 ± 14.3 24.3–63.6	46.2 ± 19.2 26.4–90.8
	Non-Trained Arm	47.3 ± 20.1 24.3–87.7	44.0 ± 11.9 27.9–59.0	46.8 ± 18.9 22.5–92.4
1RM (Kg)	Trained Arm	7.5 ± 2.5 3.5–11.0	6.3 ± 2.2 4.0–11.0	7.9 ± 2.8 4.5–13.0
	Non-Trained Arm	7.0 ± 2.8 3.5–10.5	6.6 ± 2.3 4.0-10.5	7.8 ± 3.0 4.5–14.0
MT (mm)	Trained Arm	22.3 ± 2.9 18.6–27.1	22.9 ± 4.4 15.6–31.5	22.1 ± 3.3 17.8–27.2
	Non-Trained Arm	22.1 ± 2.0 19.9–25.8	22.8 ± 4.5 12.2–27.5	21.9 ± 2.7 26.1–17.5

### Test-retest reliability of the measurements

The CV and the ICC_3, 1_ of the MVIC torque, 1-RM, and MT measurements were 4.4 ± 4.1 %, 3.0 ± 3.8 %, and 1.2 ± 0.9 %, respectively. and 0.96, 0.96, and 0.99, respectively.

### Change in MVIC torque

**Fig. 2:**
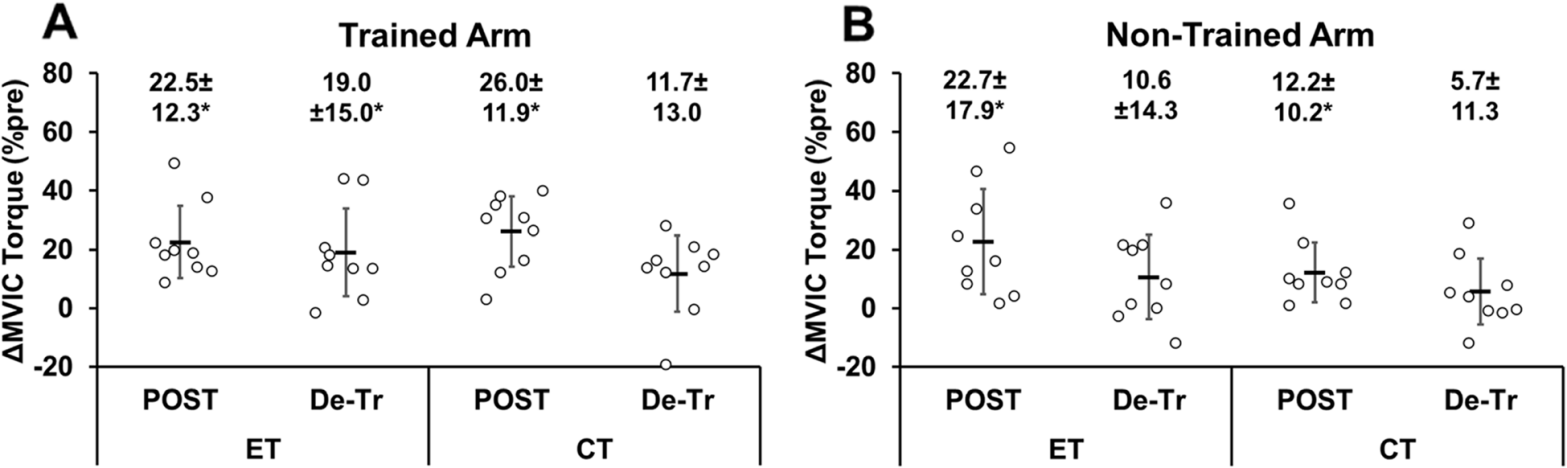
Normalized changes in maximal voluntary isometric contraction (MVIC) torque of the elbow flexors from baseline to post-training (POST) or detraining (De-Tr) for individual participants and their mean ± SD values of the eccentric training (ET, *n* = 9) and concentric training (CT, *n* = 9) groups for the trained arm (**A**) and non-trained arm (**B**) *: significantly (*P* < 0.05) different form the baseline value

MVIC torque of both arms of the control group did not change significantly before and after the 5-week period (trained arm: *p* = 0.10, Z = -1.64, non-trained arm: *p* = 0.28, Z = -1.08). MVIC torque increased from pre- to post-training in both trained and non-trained arms for the ET group (trained arm: *p* < 0.01, Z = -2.67, 22.5 ± 12.3 %, d = 0.45; non-trained arm: *p* < 0.01, Z = -2.67, 22.7 ± 17.9 %, d = 0.46), but the magnitude of increase was not significantly different between the trained and non-trained arms (*p* = 0.68, U = 35, d = 0.49). For the CT group, MVIC torque increased from pre- to post-training in both trained and non-trained arms (trained arm: *p* < 0.01, Z = -2.67, 26.0 ± 11.9 %, d = 0.69; non-trained arm: *p* < 0.01, Z = -2.67, 12.2 ± 10.2 %, d = 0.42), and the magnitude of the increase was greater (*p* = 0.02, U = 15, d = 0.84) for the trained than the non-trained arm. No significant difference in the increase in MVIC torque between groups was evident for the trained (*p* = 0.55, U = 33) and non-trained (*p* = 0.30, U = 28) arms (Fig. 2). In the ET group, MVIC torque in the trained arm remained greater (19.0 ± 15.0 %, Z = -2.55, *p* = 0.011, d = 0.38) than baseline after detraining, while that of the non-trained arm returned to the baseline after detraining (10.6 ± 15.3 %, Z = -1.72, *p* = 0.086, d = 0.23). In the CT group, MVIC torque of the trained (11.7 ± 13.0 %, Z = -1.60, *p* = 0.11, d = 0.31) and non-trained (5.7 ± 11.3 %, Z = -1.13, p = 0.26, d = 0.22) arms was not different from the baseline values after detraining.

### Changes in 1-RM strength

**Fig. 3:**
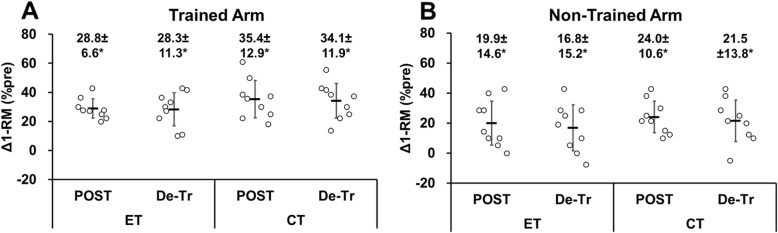
Normalized changes in one concentric repetition maximum of dumbbell curl (1-RM) from baseline to post-training (POST) or detraining (De-Tr) for individual participants and their mean ± SD values of the eccentric training (ET, n = 9) and concentric training (CT, *n* = 9) groups for the trained arm (**A**) and non-trained arm (**B**). *: significantly (*P* < 0.05) different form the baseline value

1-RM strength of both arms of the control group did not change significantly before and after the 5-week period (trained arm: p = 0.71, Z = -0.38; non-trained arm: p = 0.71, Z = -0.38)1-RM strength increased in the trained and non-trained arms for both ET (trained arm: *p* < 0.01, Z = -2.68, 28.8 ± 6.6 %, d = 0.69; non-trained arm: p = 0.01, Z = -2.54, 19.9 ± 14.6 %, d = 0.51) and CT (*p* < 0.01, Z = -2.67, 35.4 ± 12.9 %, d = 0.84; p < 0.01, Z = -2.70, 24.0 ± 10.6 %, d = 0.62) groups similarly from pre- to post-training (Fig. 3). In addition, the relative change in 1-RM strength in the ET and CT groups was not significantly different (trained arm p = 0.34, U = 29.5, non-trained arm: p = 0.44, U = 31.5). The 1-RM strength of both trained and non-trained arms was higher than the baseline values after detraining in both ET (trained arm: 28.3 ± 11.3 %, Z = -2.68, p = 0.007, d = 0.71, non-trained arm: 16.8 ± 15.2 %, Z = -2.25, *p* = 0.024, d = 0.47) and CT groups (trained arm: 34.1 ± 11.9 %, Z = -2.68, p = 0.007, d = 0.86; non-trained arm: 21.5 ± 13.8 %, Z = -2.44, *p* = 0.015, d = 0.58).

### Changes in muscle thickness (MT)

**Fig. 4:**
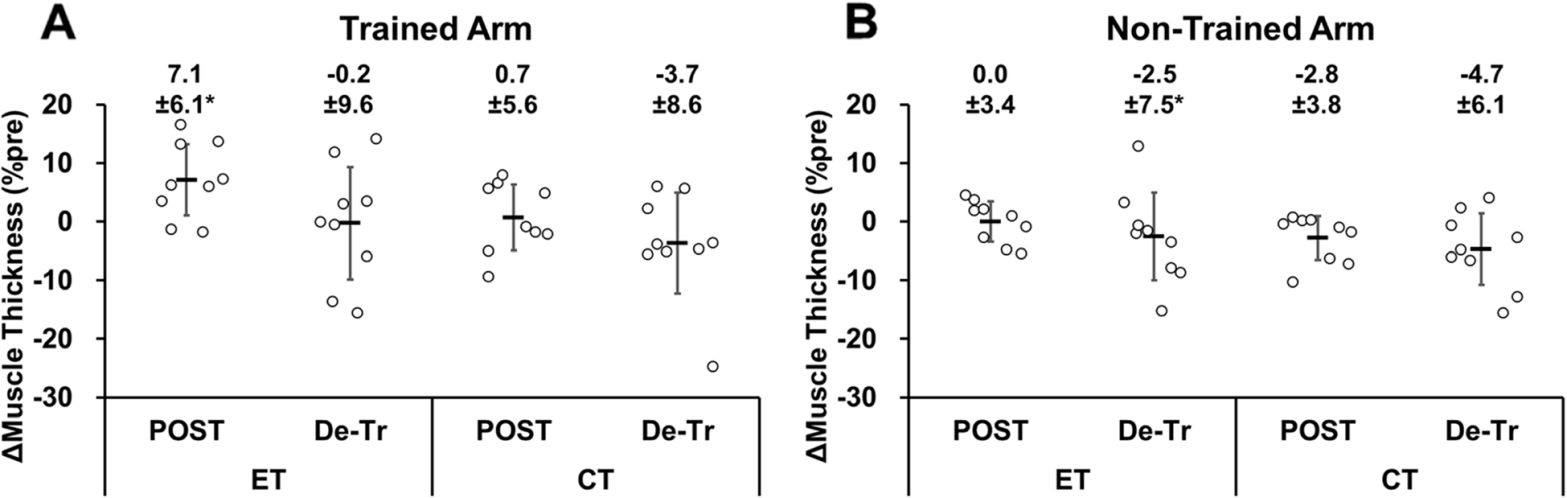
Normalized changes in muscle thickness of biceps brachii plus brachialis from baseline to post-training (POST) or detraining (De-Tr) for individual participants and their mean ± SD values of the eccentric training (ET, *n* = 9) and concentric training (CT, *n* = 9) groups for the trained arm (**A**) and non-trained arm (**B**). *: significantly (*P* < 0.05) different form the baseline value

MT of both arms in the control group did not change significantly before and after the 5-week period (trained arm: p = 0.17, Z = -2.38; non-trained arm: *p* = 0.20, Z = -1.29). MT increased (*p* = 0.02, Z = -2.31, d = 0.52) in the trained arm of the ET group only by 7.1 ± 6.1 % (Fig. 4), but it decreased and returned to the baseline value after detraining (*p* = 0.78, Z = -0.28, d = 0.05). MT did not show any changes (*p* > 0.05) over time for the non-trained arm in the ET group and the trained and non-trained arms in the CT group.

### Cross-education effect and cross-body transfer ratio

**Fig. 5:**
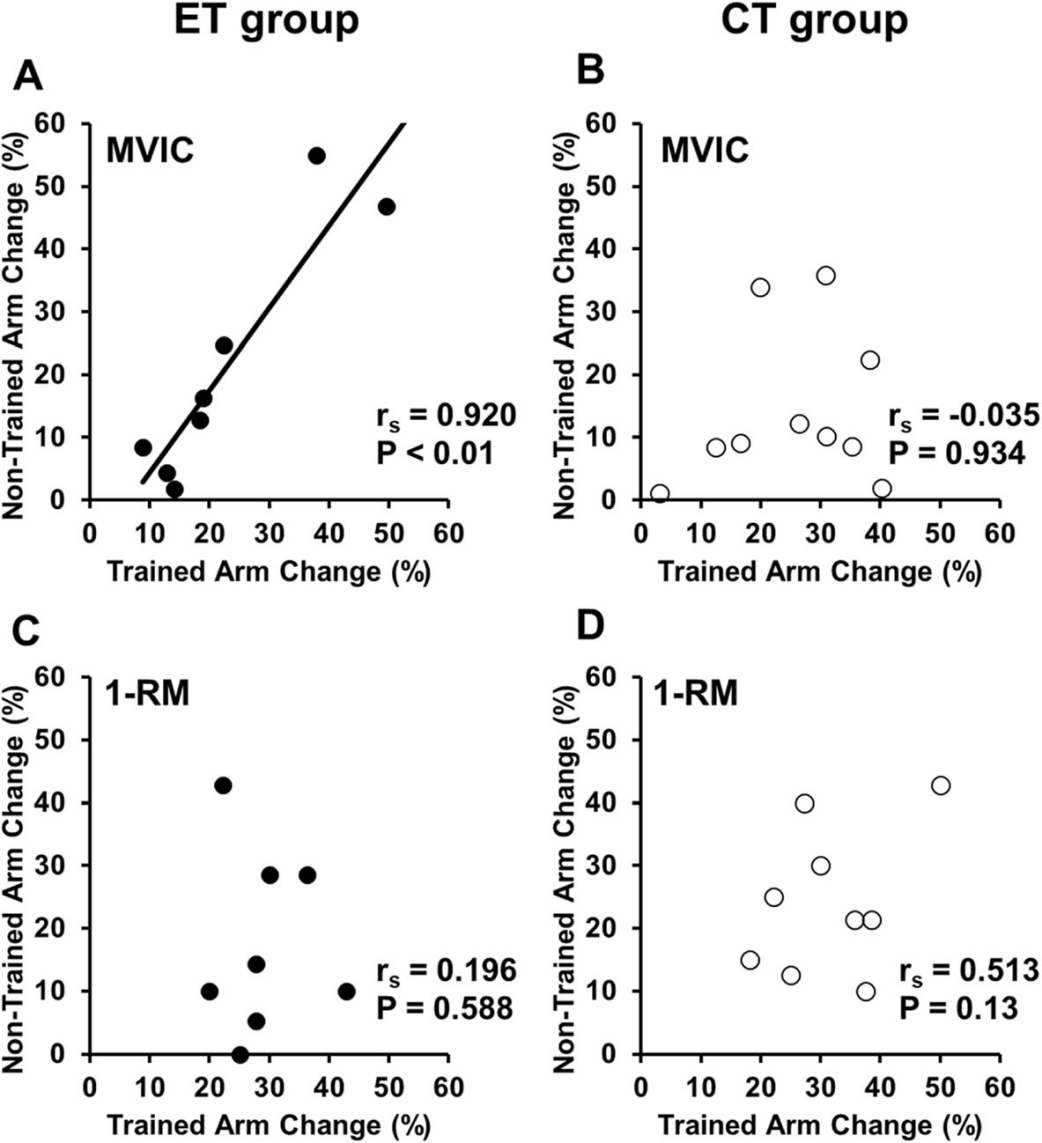
Relationships (Spearman r_s_ and p-value) between the trained arm and non-trained arm for the magnitude of changes in maximal voluntary isometric contraction torque of the elbow flexors (**A**, **B**) and one concentric repetition maximum strength (**C**, **D**) from pre- to post-training for the ET group (**A**, **C**) and CT group (**B**, **D**)

Correlations between the trained and non-trained arm for the magnitude of changes in MVIC torque and 1-RM strength from pre- to post-training are shown in Fig. 5. A positive correlation was evident for the MVIC torque (*r*_s_ =0.920, *p* = 0.01), but not for the 1-RM strength (*r*_s_ =-0.035, *p* = 0.934) in the ET group, and no significant correlation was found for the MVIC torque (*r*_s_ =0.196, *p* = 0.568) and 1-RM (r_s_ =0.513, *p* = 0.13) in CT group. For the combined data from the ET and CT groups, a significant (*p* = 0.013) positive correlation was evident for the MVIC torque (*r*_s_ =0.572), but not for the 1-RM strength (*r*_s_ =0.354, *p* = 0.149).

**Fig. 6:**
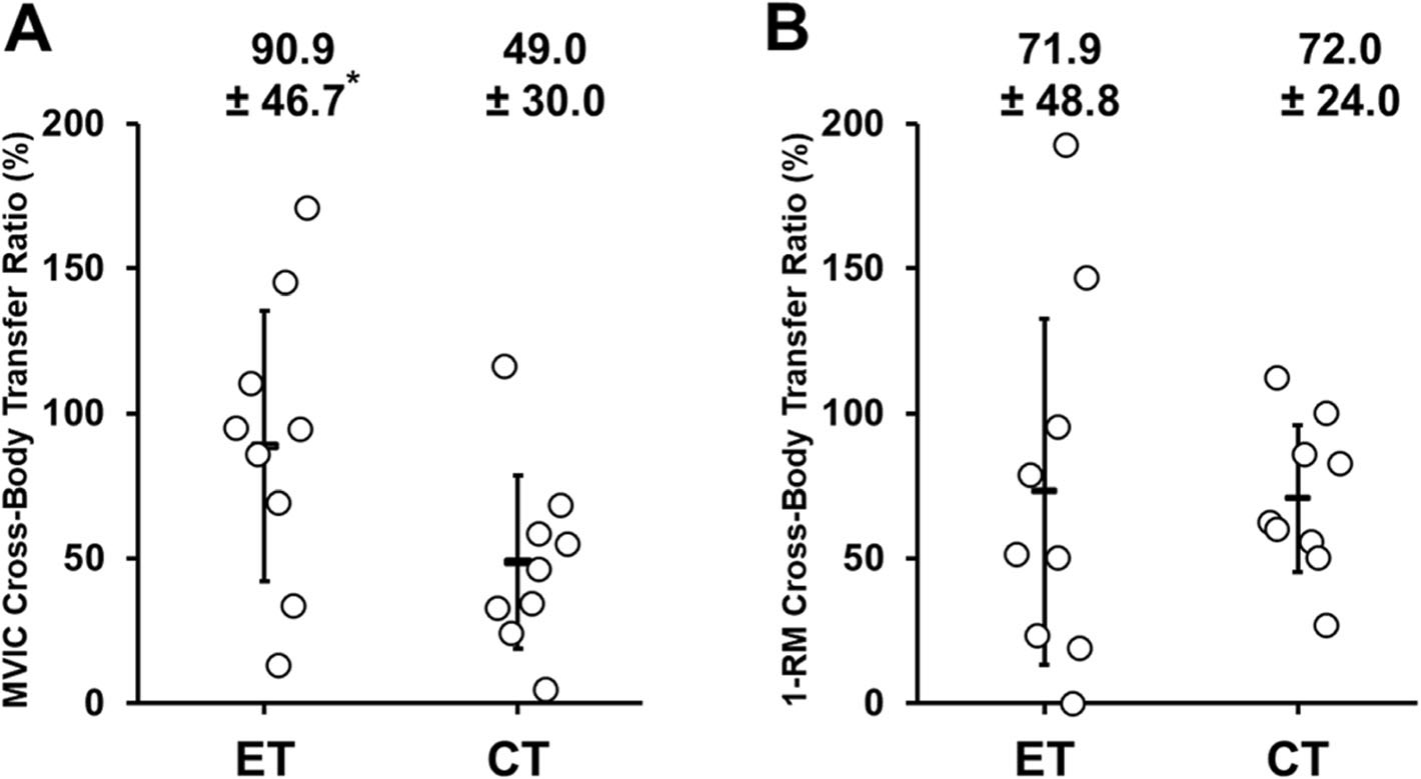
The cross-body transfer ratio from the trained arm to the non-trained arm for individual participants (shown by open circles) and their mean ± SD values (shown by the lines) of the eccentric (ET, *n* = 9) and concentric training (CT, *n* = 9) groups for maximal voluntary isometric contraction torque of the elbow flexors (**A**) and one concentric repetition maximum of arm curl (**B**). A 100% effect means that both trained and non-trained arms showed the same change. *: significantly (*P* < 0.05) different from the CT group

Figure 6 compares between ET and CT groups for the cross-body transfer ratio from the trained arm to the non-trained arm. From pre- to post-training, the ratio for MVIC torque was greater (p = 0.047, U = 18) for the ET (90.9 ± 46.7 %) than CT group (49.0 ± 30.0 %). However, no significant (*p* = 0.54, U = 33.5) difference in the ratio for 1-RM was evident between the ET (73.0 ± 59.7 %) and CT (70.6 ± 25.3 %).

## Discussion

The present study tested the two hypotheses that ET would increase muscle strength of both trained and non-trained arms and muscle thickness of trained arm greater than CT, and ET would maintain muscle strength in both trained and non-trained arms and muscle thickness of the trained arm better than CT. The results showed that (1) ET and CT increased the muscle strength of the trained arm similarly, but only ET increased the muscle thickness; (2) ET and CT increased the muscle strength of the non-trained arm similarly, but the cross-body transfer ratio of MVIC torque was significantly greater for ET than CT; and (3) after detraining, the increased MVIC toque in the trained arm was maintained after ET, but not after CT, whereas the increased 1-RM strength of the trained and non-trained arms was maintained in both groups. These results partially supported the hypotheses, but the differences between ET and CT for their training, cross-education, and detraining effects were smaller than expected.

The magnitude of increase in MVIC torque after 10 training sessions was similar between ET (22.5 ± 12.3 %) and CT (26.0 ± 11.9 %) for the trained arm (Fig. 2). It should be noted that MVIC torque was measured 12 times in total during the training period to adjust the dumbbell weight in the present study. This might have contributed to the increase in the MVIC torque from pre- to post-training. However, in the study by Tseng et al. [31], the number of the MVIC torque measures was larger (75 times) than that of the present study in the same intervention period (5 weeks) with less frequency (once a week). Although the effect of the MVIC torque measures on the increase in the torque from pre- to post-training cannot be ignored, it is likely that the effect was less in the present study than that in the study by Tseng et al. [31]. Further study is necessary to investigate the effects of MVIC measurement on muscle strength in both measurement and non-measurement arms.

Tseng et al. [31] reported a greater increase in MVIC torque after 5 sessions of ET (19 %) than CT (11 %). When comparing to the magnitude of increase in the MVIC torque found in the present study and that of the study by Tseng et al. [31], it is interesting that the magnitude did not appear to increase much by increasing the frequency for ET (22.5 %), but approximately 2-fold increase was produced for CT (26.0 %). In the present study, mild muscle soreness was observed after ET at higher intensities (e.g., 5th training session: 33.6 ± 17.4 mm). Therefore, it seems possible that the increase in MVIC torque was affected by muscle damage in ET, because the training was performed before recovery from the previous session in the ET group when a higher-intensity training session was performed. If the objective is to increase MVIC strength in a short period of time (e.g., 5 weeks), it may be better to increase the training frequency to 2 or more per week for CT, but once a week may be sufficient to increase muscle strength in ET.

Regarding the non-trained arm, a significant (*p* < 0.05) increase in MVIC torque was found after ET (22.7 ± 17.9 %) and CT (12.2 ± 10.2 %). Although the magnitude of the increase looks greater for ET than CT, due to the large variability among participants, it was not statistically significant (*p* = 0.30) (Fig. 2B). It should be noted that more participants had a 10 % or more increase in MVIC in ET (*n* = 6) than CT (*n* = 4) (Fig. 1B). Despite a similar increase in MVIC torque in the trained arm, the cross-body transfer ratio was greater for ET than CT (Fig. 6). This suggest that ET increased MVIC torque of the trained and non-trained arms more similarly than CT. Previous studies have reported a greater cross-education effect on MVIC strength after ET than CT [20, 22, 31, 32]. For example, Kidgell et al. [22] reported that after 4 weeks of unilateral resistance training of the wrist flexors performed three times a week, the increase in MVIC strength was greater in ET (43 %) than CT (11 %) for the non-trained arm. Hortobagyi et al. [20] also showed greater increases in MVIC strength of the knee extensors in the non-trained leg after ET (39 %) than CT (22 %), when the increase in the knee extensor strength of the trained leg was 45 % for ET and 36 % for CT. However, the present study did not necessarily show greater cross-education effect after ET than CT. It has been shown that high-intensity training is necessary to induce the cross-education effect [7]. For example, Carr et al. [3] reported that isometric training (5 sets of 5 repetitions for 5 s at 80 % MVIC) of the dominant elbow flexors increased MVIC strength of the non-trained dominant arm by 22 % after 5 training sessions in 2 weeks. The training intensity in the first half of the present study’s training period was low (10–30 % MVIC torque) to prevent possible severe muscle damage induced by ET. This may have attenuated the cross-education effect of ET and CT. Although the number of training sessions in the present study (10 training sessions) was twice as much as that in the study by Tseng et al. [31], it was still smaller than the minimum number of sessions required for a cross-education effect; 12 sessions, based on an isometric handgrip contraction training (Barss et al. 2018). However, it is important to note that a large cross-education effect was found in the present study even after 10 training sessions. It may be that dynamic muscle contraction training (concentric or eccentric contraction training) can induce cross-education in a smaller number of sessions when compared with isometric contraction training. Interestingly, the magnitude of the increase in MVIC torque of the non-trained arm after ET (22.7 %) and CT (12.2 %) in the present study (10 session training) was greater than that (ET: 11 %, CT: 5 %) after 5 session training reported by Tseng et al. [31]. This suggests that doubling the number of training sessions in five weeks doubled the cross-education effect in both ET and CT.

As shown in Fig. 5 a significant positive correlation was found between the trained and non-trained arm for the change in MVIC torque from pre- to post-training in the ET (r_s_ = 0.920, *p* = 0.01), but not in the CT group (*r*_s_ = -0.035, *p* = 0.934). This suggests that the magnitude of the increase in MVIC torque of the trained arm affected the magnitude of increase in MVIC torque of the non-trained arm in ET, but this was not the case for CT. A meta-analysis study by Manca et al. [24] reported a significant correlation (*r* = 0.61) between the trained and contralateral non-trained limb for changes in strength after unilateral resistance training in 31 studies, and when looking at the contraction type, the correlation was *r* = 0.55 for concentric training and *r* = 0.96 for eccentric training. It appears that the correlation was stronger for eccentric than concentric training, which was also found in the present study.

Kidgell et al. [22] reported that 4 weeks of eccentric training of the wrist flexors with maximal intensity resulted in greater reductions in both ipsilateral intracortical inhibition (32 %) and silent period duration (15–27 %) compared to concentric training of the same muscle group (2 % and 4–8 %, respectively). The authors stated that greater cortical adaptation in ET than CT contributed to the greater cross-education effect by ET. In addition, it has been reported that training on complex tasks resulted in greater cortical activation with greater bilateral connections and a greater cross-education effect than on simple tasks [13]. Eccentric contraction is considered to be a more difficult task for control of movement than concentric contraction [19]. Therefore, it was hypothesized that ET would increase muscle strength of the non-trained arm greater than CT, but no significant difference in the MVIC torque of the non-trained arm was found between ET and CT (Fig. 2). However, when comparing the cross-body transfer ratio between the trained and non-trained arms for the change in MVIC from pre- to post-training, ET (90.9 ± 46.7 %) was greater than CT (49.0 ± 30.0 %) as shown in Fig. 6. Tseng et al. [31] reported that the magnitude of the cross-education effect from the trained to non-trained arm on MVIC torque was approximately 50 % in both ET and CT groups. Although the direct comparison between studies may not be valid, the cross-education effect on MVIC torque by CT in the present study (49.0 %) was similar to that (50 %) of the finding by Tseng et al. [31], but the cross-body transfer ratio by ET in the present study (90.9 %) was greater than that (50 %) reported by Tseng et al. [31]. Since the difference between the studies was the number of training sessions in 5 weeks (10 vs. 5), it is possible that more sessions in the same period (5 weeks) enhanced the cross-body transfer ratio for ET and CT as discussed above. It is interesting that ET increased MVIC torque similarly in the trained and non-trained arms. Kidgell et al. [22] reported that after unilateral resistance training of the wrist flexors with maximal loading three times a week for 4 weeks, the magnitude of the cross-education effect on MVC strength was 44 % after concentric training but 76 % after eccentric training. As mentioned above, the authors reported greater corticospinal adaptations in eccentric than concentric training and concluded that this was a factor in the greater cross-education effect by ET than CT. It is not known if this is also the case for the elbow flexors. Further studies are warranted to investigate cortical responses (both hemispheres) to eccentric versus concentric contractions.

The increase in 1-RM was similar between the trained and non-trained arms for both ET and CT (Fig. 3), and the cross-body transfer ratio on 1-RM was not different between ET and CT (Fig. 6). This could be explained by the principle of training specificity [21]. The present study employed the concentric 1-RM test, which may have contributed more to the increased 1-RM in CT than ET. On the other hand, ET has been reported to significantly increase muscle strength in different contraction types in the trained and non-trained arms [1, 20, 22]. In fact, a similar increase in 1-RM was observed in both the trained and non-trained arms after ET and CT in the present study. Therefore, it appears that unilateral ET can cause a bilateral improvement of global muscle function.

The MT in the trained arm was significantly increased by 7.1 ± 6.1 % only after ET (Fig. 4). This suggests greater muscle hypertrophy after ET than CT. The effects of ET versus CT on muscle hypertrophy are controversial [30], but some studies have shown greater muscle hypertrophy after ET than CT [8, 12, 31], which is in line with the finding of the present study. For example, Farthing and Chilibeck [12] showed 13 % increase in MT of biceps brachii plus brachialis after the fast velocity isokinetic ET but not after CT of the elbow flexors performed three times a week for 8 weeks (24 sessions in total). In the present study, the increase in MT after ET was approximately 7 % on average, which seems to be smaller than that shown in the previous study [12]. It seems likely that the smaller increase in MT in the present study was due to the smaller number of training sessions. Regarding the non-significant increase in MT after CT, Coratella and Schena [8] also showed no muscle hypertrophy after CT and traditional (concentric and eccentric) RT. It has been reported that the expression of insulin-like growth factors and mechano-growth factors is smaller after CT than ET [18].

This was the first study to investigate the detraining effects after ET and CT. For the trained arm, the increased MVIC torque after ET was maintained over the 5-week detraining period, but this was not the case for CT, although the magnitude of increase after 5-week training was similar between ET and CT (Fig. 2). This was in line with the study by Coratella and Schena [8], showing that muscle strength was maintained during 6 weeks of detraining after ET, but not after CT and traditional (concentric and eccentric) resistance training. On the other hand, Housh et al. (1996a, b) reported that the muscle strength gained after 8 weeks of ET or CT was maintained similarly for the trained limb (ET: 100 %, CT: 93 %) after 8 weeks of detraining. However, it should be noted that Housh et al. (1996a, b) used the muscles of the lower limb that were more used in daily activities. The increased MT in the trained arm after ET returned to the baseline after detraining (Fig. 4). Thus, the longer-lasting training effect by ET than CT on MVIC torque after detraining was probably more associated with central than peripheral adaptations. It has been reported that cortical activity is greater during eccentric than concentric contractions [10]. Therefore, it seems possible that central neural adaptations contributed to the greater maintenance of MVIC torque in the trained arm post-detraining after ET than CT. However, in the present study, the increase in MVIC torque in the non-trained arm was not maintained after detraining, conflicting with the report by the previous study [16] reporting that unilateral dynamic training of the dorsi-flexors and wrist flexors performed four times a week for six weeks with 80 % MVC increased their MVC force of the non-trained limb that was maintained after six weeks of detraining. The decrease in MVIC torque of the non-trained arm by detraining in the present study in both ET and CT was probably due to the insufficient number of training sessions and training intensity, especially in the first half of the training period. It is important to note that the increased 1-RM in both the trained and non-trained arms was maintained after detraining for the ET and CT groups similarly (Fig. 3). Weir et al. (1997) reported that compared to MVIC torque, 1-RM strength was reduced less by detraining, and speculated that the smaller decrease in 1-RM strength following detraining was likely to be associated with a longer time course for the loss of skill.

The present study has several limitations. Firstly, eccentric strength was not measured in this study. Hortobagyi et al. [20] reported that ET increased eccentric strength three times more than the concentric strength gain induced by CT. Therefore, it is possible that the present study underestimated the cross-education effect of ET. Secondly, since the present study was conducted on the elbow flexors of untrained young adults, it is unclear whether similar results can be obtained in older adults and patients who need rehabilitation and in the lower limb muscle groups. Thirdly, the lack of familiarization sessions and the weekly MVIC torque measurements may have contributed to the increase in MVIC torque in trained and non-trained arms of both ET and CT groups. In the control group of this study, there was no significant change in muscle strength before and after the 5-week period, and the test-retest reliability of MVIC torque, 1-RM, and MT measurements was high (CV: <5 %, ICC: >0.95). Thus, the same number of MVIC measures should have been included to check the changes in the measures in the control group. Fourthly, the MVIC torque measures were performed by a hand-held dynamometer, although we are confident of its reliability and validity, and muscle thickness instead of muscle cross-sectional area was used to assess muscle hypertrophy. Fifthly, surprisingly, muscle strength was increased more in the non-trained arm than in the trained arm in several subjects. In the present study, it is possible that the muscle damage and fatigue caused by ET and CT, especially in the last session, remained in the post-training measurement of the trained arm. Finally, the present study did not include any measures that would shed light on the mechanisms underpinning the changes induced by the training, such as neurological or molecular measures. Future studies are necessary to understand the physiological mechanisms of adaptation in both trained and non-trained limbs.

From a practical point of view, it is important to note that increased muscle strength, especially MVIC torque of the trained arm after ET was better maintained after detraining when compared with CT. Therefore, it may be that muscle strength gained after ET is sustained better than CT to prevent muscle weakness with detraining. This is important for rehabilitation settings for the training before operation or hospitalization. The present study and previous studies [20, 22] showed that the cross-education effect of ET was greater than that of CT. This study showed that muscle strength of the trained limb and non-trained limb could be increased equally in unilateral ET compared to unilateral CT.　Prescribing ET to patients with unilateral disorders may be effective in promoting rehabilitation.

## Conclusions

The present study showed similar changes in most of the variables between ET and CT for the trained and non-trained arms, and strong cross-education effects on MVIC and 1-RM after both ET and CT, but less detraining effect on MVIC of the trained arm after ET than CT. These results did not necessarily support the hypothesis (the greater cross-education effect would be evident by ET than CT), but showed that muscle strength of the trained arm were better maintained after ET than CT in the 5-week detraining period. Further studies are warranted to investigate further the cross-education effect by ET versus CT and mechanisms underpinning the cross-education effect in training and detraining.

## Data Availability

The data collected and analysed in the present study are not publicly available due to ethical restrictions but are available from the corresponding author upon request.

## Abbreviations

CV: Coefficient variation
CT: Concentric resistance exercise training
De-Tr: Detraining
EF: Elbow flexors
ET: Eccentric resistance exercise training
ICC: Intraclass correlation coefficient
MVIC: Maximal voluntary isometric contraction
MT: Muscle thickness
POST: After training
PRE: Before training
1-RM: One-repetition maximum
